# Design and Optimization of All-Dielectric Fluorescence Enhancing Metasurfaces: Towards Advanced Metasurface-Assisted Optrodes

**DOI:** 10.3390/bios12050264

**Published:** 2022-04-21

**Authors:** Hiba Alhalaby, Maria Principe, Haitham Zaraket, Patrizio Vaiano, Anna Aliberti, Giuseppe Quero, Alessio Crescitelli, Valentina Di Meo, Emanuela Esposito, Marco Consales, Andrea Cusano

**Affiliations:** 1 Optoelectronic Division, Department of Engineering, University of Sannio, 82100 Benevento, Italy; hiba.alhalaby@unisannio.it (H.A.); principe@unisannio.it (M.P.); pvaiano@unisannio.it (P.V.); anna.aliberti@unisannio.it (A.A.); giuseppe.quero@unisannio.it (G.Q.); 2 Multi-Disciplinary Physics Laboratory, Optics and Fiber Optics Group, Faculty of Sciences, Lebanese University, Beirut 1500, Lebanon; hzaraket@ul.edu.lb; 3 Institute of Applied Sciences and Intelligent Systems, Unit of Naples, National Research Council, 80131 Naples, Italy; alessio.crescitelli@na.isasi.cnr.it (A.C.); valentina.dimeo@na.isasi.cnr.it (V.D.M.); emanuela.esposito@cnr.it (E.E.)

**Keywords:** all-dielectric metasurfaces, fluorescence enhancement, lab-on-fiber, labelled biosensing

## Abstract

The need for miniaturized biological sensors which can be easily integrated into medical needles and catheters for in vivo liquid biopsies with ever-increasing performances has stimulated the interest of researchers in lab-on-fiber (LOF) technology. LOF devices arise from the integration of functional materials at the nanoscale on the tip of optical fibers, thus endowing a simple optical fiber with advanced functionalities and enabling the realization of high-performance LOF biological sensors. Consequently, in 2017, we demonstrated the first optical fiber meta-tip (OFMT), consisting of the integration of plasmonic metasurfaces (MSs) on the optical fiber end-face which represented a major breakthrough along the LOF technology roadmap. Successively, we demonstrated that label-free biological sensors based on the plasmonic OFMT are able to largely overwhelm the performance of a standard plasmonic LOF sensor, in view of the extraordinary light manipulation capabilities of plasmonic array exploiting phase gradients. To further improve the overall sensitivity, a labelled sensing strategy is here suggested. To this end, we envision the possibility to realize a novel class of labelled LOF optrodes based on OFMT, where an all-dielectric MS, designed to enhance the fluorescence emission by a labelled target molecule, is integrated on the end-face of a multimode fiber (MMF). We present a numerical environment to compute the fluorescence enhancement factor collected by the MMF, when on its tip a Silicon MS is laid, consisting of an array of cylindrical nanoantennas, or of dimers or trimers of cylindrical nanoantennas. According to the numerical results, a suitable design of the dielectric MS allows for a fluorescence enhancement up to three orders of magnitudes. Moreover, a feasibility study is carried out to verify the possibility to fabricate the designed MSs on the termination of multimode optical fibers using electron beam lithography followed by reactive ion etching. Finally, we analyze a real application scenario in the field of biosensing and evaluate the degradation in the fluorescence enhancement performances, taking into account the experimental conditions. The present work, thus, provides the main guidelines for the design and development of advanced LOF devices based on the fluorescence enhancement for labelled biosensing applications.

## 1. Introduction

Metamaterials are subwavelength structured materials engineered to exhibit unique optical properties that are not commonly available in natural materials [1,2]. Such artificial materials have received attention from researchers, and they allowed to attain extraordinary light phenomena such as negative refraction [3], super lensing [4], and complete control of the electromagnetic fields [5]. Recently, the two-dimensional counterpart of metamaterials, known as metasurfaces (MSs), has gained increasing attention due to their simpler fabrication using common techniques, such as photolithography and electron-beam lithography, and on-chip and on-fiber assembling. A MS is an ultrathin planar optical element composed of resonating elements that exhibits the ability to control light at the nanoscale. By properly designing those nanoantennas, it is possible to mold at will an impinging electromagnetic field, thus enabling flat-optics and photonics [6,7]. MSs have been reported in various applications, including wavefront shaping [8,9,10], dispersion engineering [11,12], nonlinear optics [13], communications [14], sensing [15,16,17,18,19], and microscopy [20].

In 2017 a pioneering work by Principe et al. [21] showed the first integration of a MS with an optical fiber, giving birth to the optical fiber meta-tips (OFMT). The integration of MSs with optical fibers represents a valuable addition to the growing field of “Lab-on-fiber” (LOF) technology [22,23,24], endowing optical fibers with the extraordinary capabilities of MSs that might be crucial to improving the performances of LOF-based devices. Furthermore, OFMTs are particularly apt for miniaturization and integration with plug-and-play devices [25,26]. First, the OFMTs presented in [21] were based on plasmonic phase gradient MSs made of inhomogeneous arrays of rectangular aperture nanoantennas milled in a thin gold layer, previously deposited on the fiber end-face. The proof-of-concept application was the beam steering of a transmitted beam by an arbitrary deflection angle. It was also shown that OFMTs deflecting the output beam by 90° are able to efficiently couple normally incident light to surface waves and exhibit a sensitivity to local refractive index variations higher than that of the corresponding gradient-free uniform array [21,27,28].

More recently, using the well-established biotin–streptavidin pair as a benchmark, Consales et al. demonstrated the capability of a suitably designed MS-enhanced LOF biosensor to significantly outperform standard plasmonic biosensors in real biological experiments [27,28]. In the same work, they also demonstrated the capability of the realized MS-assisted label-free optrode to detect Streptavidin in running buffer solutions with a limit of detection as low as 3 ng/mL [28]. 

**Figure 1:**
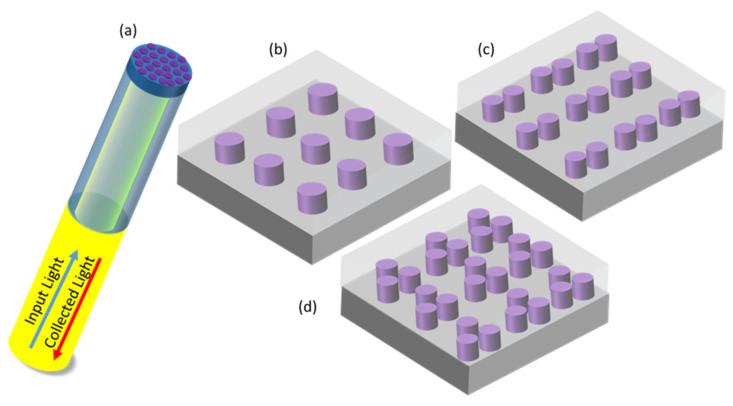
Schematic illustration of the studied geometries. (**a**) A schematic of a fiber with NSs fabricated on its tip. A square-periodic array of: (**b**) cylindrical Si NSs (**c**) dimers of Si cylindrical NSs and (**d**) trimers of cylindrical Si NSs on SiO_2_ substrate in a homogeneous medium with a refractive index = 1.33.

With the aim of further improving the sensing performances of LOF biosensors, in this paper, we explore the possibility of developing advanced MS-assisted fluorescence (FL)-based optrodes. To this aim, with reference to the schematic illustration reported in Figure 1a, the integration on the fiber tip of properly designed MSs able to enhance the fluorescence signal emitted by a target molecule would allow the realization of a label-based biosensor characterized by very low limits of detection, thanks to the FL-intensity enhancement. This would, in turn, represent a valuable addition to the arsenal tools available in the LOF technology. 

So far, several papers have demonstrated FL-intensity enhancement up to three orders of magnitude exploiting plasmonic MSs [29,30,31,32]. Metal enhanced fluorescence [33,34] is well established and is of importance to many research areas reviewed in [35]. However, plasmonic materials suffer from high dissipation losses causing heat generation in the structure, which can be a limitation in many applications [36,37]. Besides, owing to the high absorbance of metals, a quantum emitter positioned in the vicinity of a plasmonic nanostructure (NS) undergoes quenching, which makes dielectric spacers essential between the emitter and the NS, compromising the field enhancement near the NS [38,39].

For these reasons, high-index dielectric NSs have been explored as an alternative to plasmonic metal nanoantennas [40,41,42]. Sub-wavelength dielectric NSs support Mie resonances, producing enhanced field intensity in their proximity [43]. Moreover, high-index materials feature a large electron bandgap and thus weaker absorption losses in the visible and infrared regimes. Importantly, while metal NSs feature only electric modes and need complex geometries to acquire resonance of a magnetic nature, dielectric NSs support both electric and magnetic resonances [44,45]. NSs featuring spectral overlap of electric and magnetic dipole resonance offer the opportunity to achieve the desired directionality with a more compact design [46], as predicted a few decades ago by Kerker et al. [47]. Thus, dielectric nanoantennas have a great potential to enhance the fluorescence intensity by improving the excitation and emission rates of a fluorophore and controlling its emission directivity [48]. Several papers have been published in the last years exploiting dielectric NSs for several applications, including fluorescence enhancement [49,50,51,52,53,54,55], Raman scattering [50], and biosensing [56]. A comparative experimental study between dielectric silicon and plasmonic dimers shows how the former avoids any parasitic heating and quenching [51,57]. In [58], a fluorescence enhancement factor exceeding 1000 has been experimentally demonstrated by employing an array of silicon cylindrical disks featuring Mie resonances. Fluorescence enhancement up to 3600 was also achieved using gallium phosphide (GaP) nanoantennas [53]. Moreover, FL-sensing platforms based on all-dielectric FL biosensors were able to directly detect a target antibody at very small concentrations (orders of femtomolar) [59] and to detect nucleic acid targets [60], proving that FL biosensors based on all-dielectric MSs exhibit high performance. 

Combining the advantages of all-dielectric MSs with fiber optic technology, we envision a high sensitivity miniaturized platform for labeled biosensing applications, in which a single fiber probe can be used to detect biological targets up to very low concentrations thanks to the fluorescence-intensity enhancement. The fiber optics technology will allow the development of a point of care device and decrease the complexity of the optical setup by using the fiber optic as a light coupled substrate that illuminates the sample under analysis and simultaneously collects the emission from the FL molecules labeling the biological target. 

A substantial quantity of work on planar substrates consisting of all-dielectric MSs for fluorescence enhancement already exists; however, to the best of our knowledge, no work has considered optical fibers as substrate for fluorescence-based labelled biosensors. With the final goal of realizing an advanced labeled LOF biosensor based on the integration of FL-enhancing all-dielectric MSs, we designed resonant dielectric structures to be realized on the end face of optical fibers, capable of enhancing fluorescence emission and collection when operating in reflection mode. Specifically, here we present a comparative numerical study of FL enhancement of emitters when coupled to different optical MSs, made of periodic arrays of silicon cylindrical NSs, or of dimers or trimers of cylindrical nanoantennas. The period of the arrays is chosen to match the work wavelength (which coincides with the NSs resonance wavelength) to the first Rayleigh-Wood anomaly [61,62]. As a result, the localized excitation of the individual NSs and the surface lattice resonance interfere, producing narrow spectral features and significantly enhanced field intensities in the proximity of the NSs [63]. Using the commercial software Comsol Multiphysics based on the Finite element method (FEM), we compare the three different geometries numerically and study their influence on enhancing the fluorescence emission of emitters, by assuming that such MSs lie on the tip of a 200 μm-core MMF, which is used both as the excitation source and as the collective probe. An efficient collection of the emitted photons is also necessary to improve the ultimate sensitivity; indeed, the effect of light redirection induced by the nanoantennas will improve the number of collected photons by the MM optical fiber, whose numerical aperture is taken into account in the numerical study.

## 2. Materials and Methods

### 2.1. Structure and Method

In the following, we will study how a periodic array of various dielectric structures affects the fluorescence emission enhancement from an emitter. Three dielectric geometries that consist of silicon NSs are considered: (i) the simplest case was an array of single nanocylinders; then (ii) more complex structures were studied, made of dimers with 20 nm-gap; and (iii) trimers of cylindrical NSs. These NSs, supported by SiO_2_ substrate (i.e., the fiber end-face) and embedded in a medium, were designed to maximize the collected FL signal at the wavelength of 650 nm. 

The refractive index of amorphous silicon (a-Si) in the wavelength range of interest was evaluated from ellipsometry measurements of a layer deposited on a glass substrate via e-beam evaporation and that of SiO_2_ is set to 1.45 and the medium to 1.33 neglecting their dispersion. The field maps, and the computation of the fluorescence enhancement factors, were performed using the RF module based on the finite element method (FEM) implemented in the commercial software Comsol Multiphysics [64]. For the computation of the reflectivity spectrum, the rigorous-coupled wave analysis (RCWA) is also adopted [65], to double-check the results.

The unit cell includes the silicon nanoantenna with periodic boundary conditions applied in the x and y directions. Ports were assigned to the upper and lower boundaries in the z-direction to illuminate the structure with a normal incident plane wave linearly polarized, traveling along z. Indeed, it is assumed that most of the power will be delivered to the fundamental mode, which has a (approximately) Gaussian transverse profile that behaves locally (within the unit cell) as a plane wave [66].

An adaptive meshing is used with a maximum element size of λ_m_/10 and a minimum element size of λ_m_/15, where λ_m_ is the wavelength traveling in the domain. The diameter (D) and height (h) of the cylindrical antenna were varied to produce Mie resonances in the visible range while the period (Λ) is fixed. A parametric study is carried out in order to obtain resonance at the maximum absorption wavelength of the intended dye molecule. 

### 2.2. Fluorescence Enhancement Factors 

The fluorescence enhancement intensity fl of the emitters coupled to the NSs is computed as the product of the excitation rate enhancement ($\gamma_{\mathrm{exc}}$), quantum yield ($\frac{\eta }{\eta_{0}}$), and collection efficiency enhancement $\left(\frac{\kappa_{\mathrm{coll}}}{\kappa_{\mathrm{coll}}^{0}}\right)$ [67]

(1) $$\mathrm{fl}=\gamma_{\mathrm{exc}}*\frac{\eta }{\eta_{0}}*\frac{\kappa_{\mathrm{coll}}}{\kappa_{\mathrm{coll}}^{0}}$$

**Figure 2:**
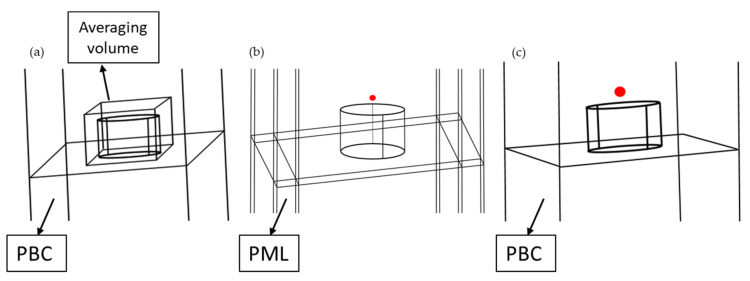
Schematic representation of geometries used for computing the three factors separately. (**a**) Square volume surrounding the NS to compute the average electric field enhancement with Floquet-type periodic boundary conditions (PBC) around. (**b**) Dipole above the nanostructure surrounded with perfectly matched layers (PMLS) to compute the quantum yield enhancement of the fluorophore. (**c**) Dipole above the NS, to compute the far field radiation pattern.

The resonant NSs will create near-field enhancements which improve light-matter interaction resulting in a boosted excitation rate. The excitation rate enhancement of the fluorophore $\gamma_{\mathrm{exc}}\;$ placed in the vicinity of an optical antenna is defined as [68]:

(2) $$\gamma_{\mathrm{exc}}=\frac{{\left|p\;.\;E\right|}^{2}}{{\left|p\;.{\;E}_{0}\right|}^{2}}$$

where p is the dipole moment modeling the fluorophore, E and E_0_ are the electric field in the presence and in the absence of the NSs, respectively, at the dipole location. In our numerical analysis, the electric field is computed in a single point at a specific distance from the NS, or its average value is computed inside a square volume surrounding the NS where the fluorophores are expected to be (Figure 2a).

After excitation, the fluorophore can return to its ground state, emitting a photon at a longer wavelength λ_em_ or decaying non-radiatively. This phenomenon is characterized by the quantum yield of the fluorophore. In a homogeneous medium, the intrinsic quantum yield, a property of the dye, is defined as $\eta_{0}=\frac{\gamma_{r}^{0}}{\gamma_{r}^{0}+\gamma_{\mathrm{nr}}^{0}}$ where $\gamma_{r}^{0}$ and $\gamma_{\mathrm{nr}}^{0}$ are, respectively, the radiative and non-radiative decay rates of the molecule in free space. The decay rates of an emitter coupled to an antenna are altered when the resonant wavelength is tuned to overlap the emission wavelength leading to a change in its quantum yield as [69]

(3) $$\frac{\eta }{\eta_{0}}=\frac{\gamma_{r}/\gamma_{r}^{0}}{\eta_{0}\left(\left(\gamma_{r}+\gamma_{\mathrm{nr}}\right)/\gamma_{r}^{0}\right)+\left(1-\eta_{0}\right)}$$

The factors $\frac{\gamma_{r}}{\gamma_{r}^{0}}\;$ and $\frac{\gamma_{\mathrm{nr}}}{\gamma_{r}^{0}}$ are computed numerically as $\frac{\gamma_{r}}{\gamma_{r}^{0}}=\frac{P_{r}}{P_{r}^{0}}\;$ and $\frac{\gamma_{\mathrm{nr}}}{\gamma_{r}^{0}}=\frac{P_{\mathrm{abs}}}{P_{r}^{0}}$ where $P_{r}$ is the radiated power by the molecule coupled to the NS, $P_{r}^{0}$ is the radiated power by the emitter in a homogeneous medium (in absence of the NS), and $P_{\mathrm{abs}}$ is the power absorbed by the nanoantenna [70,71,72]. An oscillating electric dipole representing a fluorophore is used inside a finite structure to calculate the emission enhancement from an emitter when coupled to NSs [69,72]. Perfectly matched layers are used in the x, y, and z boundaries, including the NS and the dipole (Figure 2b). The radiated power $\left(P_{r}\right)$ from the dipole is computed by integrating the energy flux on a sphere closely surrounding the dipole. The dimension of the sphere, which does not affect the calculated power, was chosen to avoid any intersection with the nanoantenna structures. The absorbed power ($P_{\mathrm{abs}}$) by the nanoantenna is computed by integrating the losses over the nanoantenna (Figure 2c).

The directionality properties of the emitter coupled to the NSs have been studied. This is a crucial aspect for our purpose since the MMF has a limited numerical aperture with a fixed orientation. Thus, the designed MS must also be able to redirect photons into the acceptance angle of the optical fiber.

The 3D scattering patterns is further processed in Matlab to compute the collection efficiency ($\kappa_{\mathrm{coll}}$) of the system that was computed as follows [70]

(4) $$\kappa_{\mathrm{coll}}=\frac{\int_{0}^{2\pi }\int_{0}^{\theta_{\max}}\mathrm{Pr}\left(\theta ,\varphi \right)\sin\left(\theta \right)d\theta d\varphi }{\int_{0}^{2\pi }\int_{0}^{\pi }\mathrm{Pr}\left(\theta ,\varphi \right)\sin\left(\theta \right)d\theta d\varphi }$$

Thus, only light emitted in the acceptance cone can contribute to a measurable signal by a setup with a numerical aperture $\mathrm{NA}=n*\sin\;\left(\theta_{\max}\right)$, where $\theta_{\max}$ is the maximum acceptance angle range of the collection objective and n is the refractive index of the medium. Note that to take into account the effect of the periodic array designed, array factor multiplied by the power emitted to the far-field Pr(θ,φ) is considered in the above calculation. Array factor (AF) for N × M elements in planar geometry (2D) along x and y is calculated as follows [73]:

(5) $$\begin{array}{c} \mathrm{AF}=\overset{N}{\underset{n=1}{\sum }}\overset{M}{\underset{m=1}{\sum }}a_{\mathrm{nm}}e^{{\mathrm{jk}\psi }_{\mathrm{nm}}\left(\theta ,\varphi \right)}\\ \psi_{\mathrm{nm}}\left(\theta ,\varphi \right)=x_{n}\sin\left(\theta \right)\cos\left(\varphi \right)+y_{m}\sin\left(\theta \right)\sin\left(\varphi \right) \end{array}$$

For equally spaced arrays: along the *x*-axis ${\;x}_{n}=n.d$, along the *y*-axis ${\;y}_{m}=m.d$, where d is the distance between elements, $a_{\mathrm{nm}}\;$ is the array coefficients or weights, which are all equal in our case.

## 3. Results

### 3.1. Optical Properties of Investigated Structures

**Figure 3:**
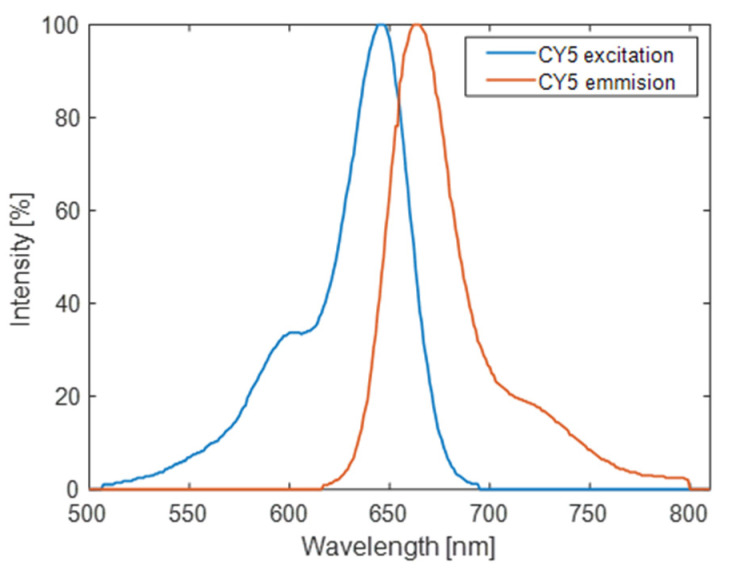
Excitation and emission spectra of cyanine-5 fluorophore. Spectral data obtained from Thermofisher SpectraViewer [75].

**Figure 4:**
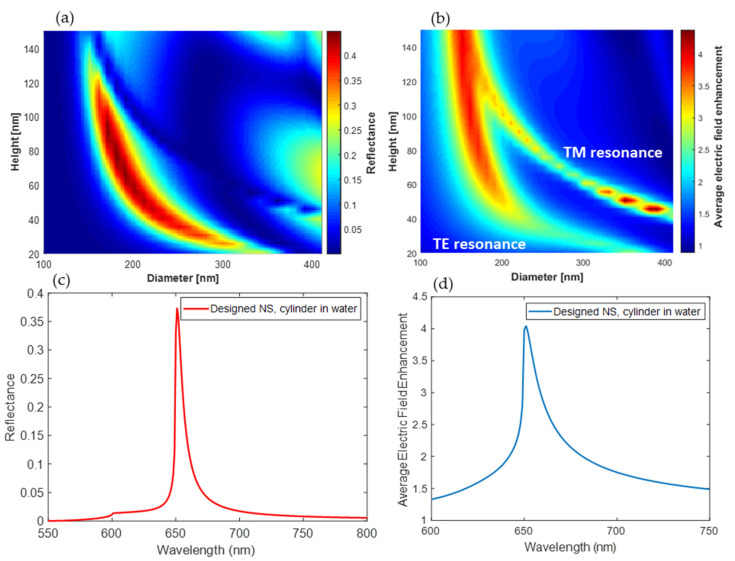
Optical properties of the inspected structures. (**a**) Computed reflectance and (**b**) average electric field enhancement E/E0 in a domain surrounding the nanoantenna as a function of the dimensions of the nanoantenna. (**c**) Computed reflectance and (**d**) average electric field enhancement spectra of the designed NS: 448 nm period array of a cylinder NS with the chosen parameters; diameter = 160 nm and height = 120 nm.

We are interested in resonance around λ = 650 nm, which correspond to the maximum excitation wavelength of the fluorophores we consider (i.e., the cyanine 5, whose excitation and emission spectra are shown in Figure 3). However, this study can be applied on a wide range of different fluorophores, and the dimensions of the NSs can be modified accordingly to have the resonance at the required wavelength. The scattering from dielectric NSs generates strong electric and magnetic resonant modes as described in Mie theory. The existence of electric and magnetic dipole resonance depends on both the height and the diameter of the cylindrical NSs with a defined refractive index [74]. Thus, to choose the geometrical dimensions of our design that will result in a resonant behavior, the height (h) and diameter (D) were varied, while fixing the period and wavelength at 448 nm and 650 nm, respectively. The period (Λ) of the structure is equal to the resonance wavelength in the medium (λ = nΛ) to exploit the interference between lattice surface mode and the antenna’s resonance. This results in a resonance characterized by narrow spectral line cuts and increased field intensities in the proximity of the NS array [63]. The resulting color-map (illustrated in Figure 4) clearly shows two resonance branches for diameter values ranging between 100 and 400 nm and height values ranging between 20 and 150 nm. The local electric field enhancement E/E_0_ is averaged in a square domain surrounding the nanoantenna at a distance of 15 nm which represents the volume where the molecules are expected to spread.

**Figure 5:**
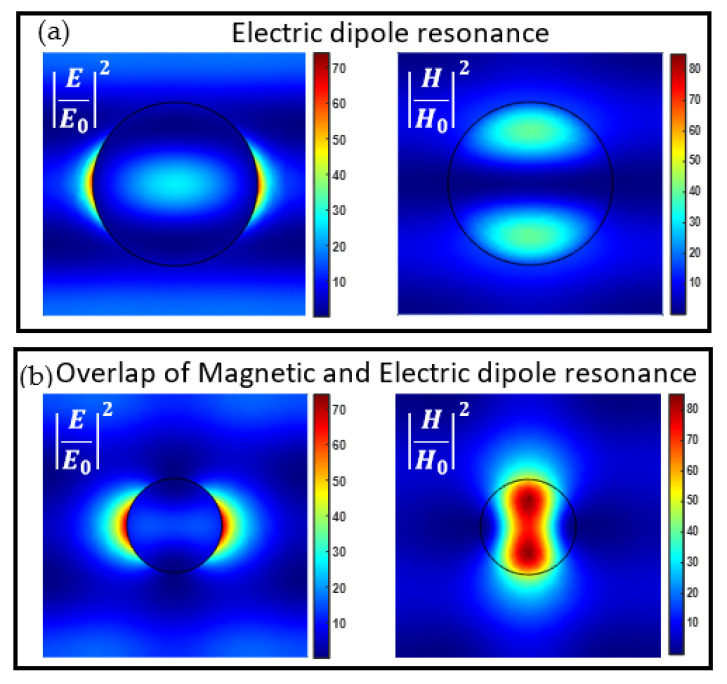
Electric and magnetic field maps at λ = 650 nm in a cut plane through the center of the NS (circle in black) for (**a**) a resonant cylinder (D = 200 nm, h = 60 nm) featuring electric dipole resonance and (**b**) a resonant cylinder (D = 160 nm, h = 120 nm) featuring an overlap of the electric and magnetic dipole resonance.

In Figure 4, the numerical averaged electric field enhancement and the reflectance are shown at λ = 650 nm. In both maps, it is possible to identify the electric and magnetic resonances. From these maps, we choose the most convenient design in order to maximize the electric field enhancement and the photons collection (see Section 3.3). The complete overlap of the two resonances yields an almost flat reflectivity spectrum [46], however, having a peak in reflection is convenient for the characterization and validation of the fabricated sample. Hence, we chose a configuration where the partial overlap of the transverse electric (TE) and transverse magnetic (TM) resonances is enough to guarantee a high electric field enhancement and an asymmetric radiation diagram of a dipole in close vicinity (as shown in Section 3.3). The chosen parameters are: h = 120 nm and D = 160 nm. A resonance peak is shown at λ = 650 nm in the reflectance spectrum, and a peak in the average electric field enhancement. In order to clarify the TE and TM resonances overlap, we define a cylinder height-diameter aspect ratio as X = h/D and study the electric and magnetic fields for different values of X. In accordance with reference [46], for X > 0.5 we have an overlap of electric and magnetic dipole resonance while for X < 0.5 the structure will feature non-degenerate electric and magnetic dipole resonances. As an example, in Figure 5 we study the behavior of a periodic array of cylindrical nanoantennas with the parameters h = 120 nm and D = 160 nm having an X = 0.75, and cylindrical nanoantennas with the parameters h = 60 nm and D = 200 nm, having lower aspect ratio X = 0.3. The electric and magnetic field maps at λ = 650 nm in a cut plane through the center of the nanoantenna are shown in Figure 5. The resonance with the lower aspect ratio (X < 0.5) exhibits electric dipole mode characteristics, with the electric field profile showing an antinode and the magnetic field profile showing a node at the center. On the contrary, for X > 0.5 there exists an overlap of electric and magnetic dipole resonance, as can be seen from the electric and magnetic field maps.

Similar calculations have been performed for the dimer and trimer of cylindrical Si nanoantennas. Although smaller gaps might yield larger field enhancement [51], a gap of 20 nm was chosen in consideration of the fabrication limits and of the space needed for the intended biological target to reach the maximum localized enhancement field inside the gap.

**Figure 6:**
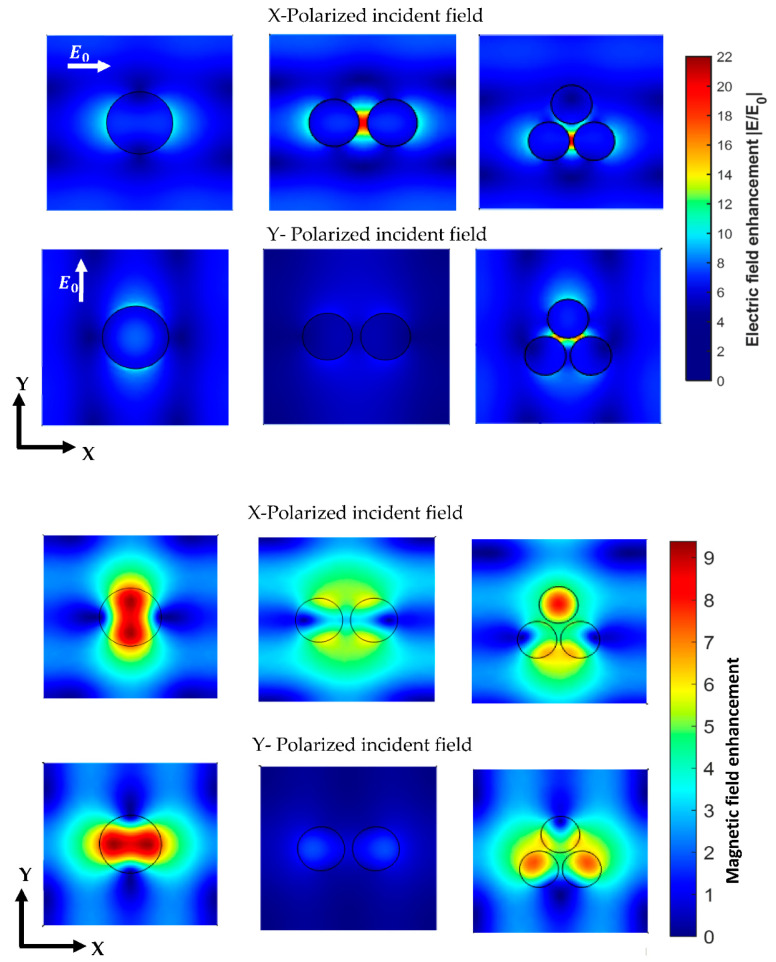
Simulated field maps of the electric and magnetic intensity distribution enhancement in the plane located at the center of the NSs. The incident field is excited at λ = 650 nm.

The diameter and height of the cylindrical NSs that exhibit a resonant behavior at the desired wavelength are D = 120 nm and h = 85 nm in the dimer configuration, and D = 100 nm and h = 105 nm in the trimer configuration. The field enhancement in the vicinity of the nanoantenna can reach a value of 20 in the dimer and trimer cases, which is about two times higher than the value obtained in the case of single cylindrical NSs. In the trimer configuration, where the three cylinders are positioned at the vertices of an equilateral triangle, the presence of a third cylinder does not improve the electric field enhancement with respect to dimer configuration, but yield a good field enhancement for both incident polarizations. This is demonstrated in the field maps in Figure 6, where it is evident that the electric field is maximized around the NS in the case of a single nanoantenna and inside the gaps in the dimer and trimer configurations. 

The influence of the incident polarization on the field enhancement is highlighted in the studied geometries. The maximum electric field enhancement is not affected by the incident polarization in the symmetrical case (the cylindrical configuration). However, a transverse polarization does not yield any significant enhancement in the nanogap of a dimer configuration. Thus, the dimer nanoantennas will result in a field enhancement in the nanogap when the incident polarization is along the dimer direction. As for the trimer configuration, the electric field enhancement will be localized in the gap between the base NSs and the central one when the incident field is *Y*-polarized, recording a maximum field enhancement of about 15.

### 3.2. Resonant-Driven Excitation and Emission 

**Figure 7:**
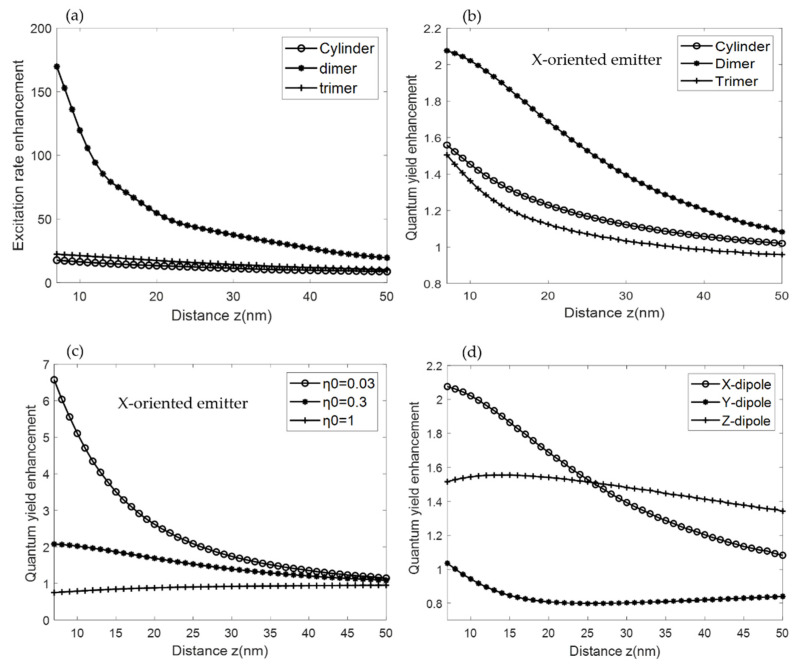
Excitation and emission enhancement as a function of distance z (nm). (**a**) Excitation enhancement and (**b**) quantum yield enhancement variation of a fluorophore with an *x*-orientation and intrinsic quantum yield η_0_ = 0.3 when coupled to different geometries. (**c**) Quantum yield enhancement variation with distance z (nm) for fluorophores having intrinsic quantum yield η_0_ = 0.03, 0.3, 1 when coupled to dimer nanoantenna. (**d**) Quantum yield enhancement variation of a fluorophore with *X*, *Y*, and *Z* orientation with an intrinsic quantum yield η_0_ = 0.3 coupled to dimer nanoantenna.

In the following, we compared the contributions of the three factors described in Section 2.2 for the designed arrays of the single, dimer, and trimer cylindrical NSs supporting resonances. The enhancement of the excitation rate $\;\frac{\gamma_{\mathrm{exc}}}{\gamma_{\mathrm{exc}}^{0}}$ is obtained from the calculated electric field enhancement at λ = 650 nm by using Equation (1). The variation of the excitation rate enhancement with the distance z above the three different structures is depicted in Figure 7a. Maximum excitation rate is present at the NSs surface and decreases when increasing the distance z. The dimer structure resulted in the maximum excitation rate enhancement of 170, an order of magnitude higher than that of single cylinder and trimer structures. This is explained by the “hot spot” localized in the gap of the dimer NSs and could be of great advantage for enhancing the excitation of a single fluorophore with the possibility of controlling its position precisely.

Figure 7b–d illustrates the quantum yield enhancement variation as a function of the distance z between the single emitter and the structures. The position of the emitter is centered above the structure with an intrinsic quantum yield η_0_ = 0.3 in a homogeneous medium. The results show that the quantum yield of a fluorophore is altered when coupled to the structures. This is due to the modification of the fluorophore decay channels when coupled to a resonant structure. Since dielectrics have significantly reduced absorption compared to plasmonics, quantum yield is enhanced even at distances as low as 7 nm. This will eliminate the need to use dielectric spacers taking advantage of the maximum enhanced field in the vicinity of the structures with negligible ohmic losses. This enhancement will also strongly depend on the intrinsic quantum yield of the emitter and its orientation. This has been illustrated in Figure 7c where an *x*-oriented dipole is coupled to the dimer structure considering lower intrinsic quantum yield values. A 6.5-fold enhancement of quantum yield is achieved for an emitter with η_0_ = 0.03 centered at z = 7 nm above the structure. For higher intrinsic quantum yield fluorophores, a lower enhancement of about 2 is obtained. Moreover, this enhancement will change with the orientation of the emitter (Figure 7d), an emitter with a *Y*-orientation coupled to dimer NS will have almost 50% lower quantum yield enhancement than the *X*-oriented dipole at low distance. Thus, the quantum yield enhancement will critically depend on the intrinsic quantum yield of the emitter and its relative position to the structure.

An efficient collection of the emitted photons is also needed for good performance. Coupling an emitter to a resonant structure will break the symmetrical scattering of light, and a controlled directivity is achieved [76]. The effect of light redirection will improve the number of collected photons by optical instruments having a limited NA. In the following, we considered an array of cylindrical NSs on the tip of a MMF with NA = 0.5.

### 3.3. Collection Efficiency Enhancement 

**Figure 8:**
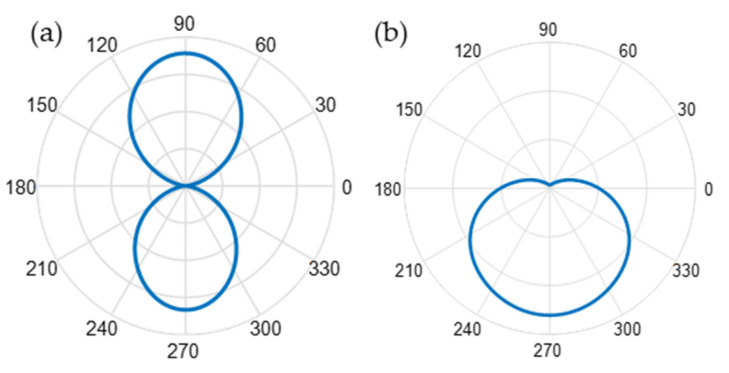
Cross-section of the far-field radiation pattern of an *x*-oriented dipole when coupled to (**a**) a reference structure including a glass surface and (**b**) resonant cylindrical structure featuring an overlap between electric and magnetic dipole resonances.

The illumination of the molecules and the collection of their emission are assumed to be both through the same optical fiber. The partial overlap of electric and magnetic dipole modes in the spectrum of this resonant structure results in obtaining higher emission in the desired direction (towards the optical fiber end-face). This controlled directivity will enhance the collection efficiency of the optical fiber as defined in the above section. The computed radiation pattern of the emitted light intensity to the far-field P(θ,φ) is illustrated in Figure 8. The dipole oriented along x is emitting symmetrically in Figure 8a when positioned in water above the glass, while the dipole will emit in the direction of the optical fiber when coupled with the resonant structure. To quantitatively analyze the enhancement in terms of directivity of the emitters when coupled to periodic silicon NSs, the radiation diagram of the MS, and the collection efficiency of the optical fiber with an acceptance angle θ_max_ = 22° have been computed according to Equation (4). Table 1 shows the collection efficiency enhancement of the setup for the three different orientations of a dipole coupled to the different considered structures. The dipole is located at 7 nm above the structures. For an array of cylinders coupled with *X* and *Y*-oriented emitters, the same collection efficiency enhancement is recorded due to the symmetry of the structure, while in the *Z*-oriented dipole case, the collection efficiency enhancement will diminish. In the case of an array of dimers and trimers, the collection efficiency enhancement is maximum when the dipole is parallel (*X*-oriented) to the dimer direction, this enhancement decreases when the dipole is perpendicular to the dimer direction (*Y*-oriented dipole). The presence of the third cylinder in the trimer case will result in a significant increase in the collection efficiency enhancement when coupled with a *Z*-oriented dipole.

## 4. Discussion

### 4.1. Overall Fluorescence Enhancement with Resonant Dielectric MSs

**Figure 9:**
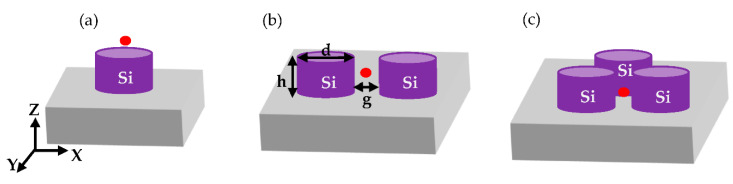
Schematic illustration of: (**a**) cylinder NS on SiO_2_ substrate with a point dipole, which represents a fluorophore situated above the NS; (**b**) dimer NSs; and (**c**) trimer NSs on SiO_2_ substrate with a point dipole situated in the gap.

In this section, we compute the overall fluorescence enhancement (fl) of an emitter with an intrinsic quantum yield η_0_ = 0.3 when coupled to the designed structures. We study different locations and orientations of the dipole. For the single cylinder MS, the emitter is centered 7 nm above or aside the nanoantenna; for the dimer and trimer configurations, the emitter is centered 7 nm above or in the gap. An *x*-directed and a random orientation are considered (Figure 9). The calculation is carried out at the maximum excitation wavelength of 650 nm. Table 2 and Table 3 demonstrate the improvement of the fluorescence signal provided by the periodic arrays of the dielectric structures in different cases. The array of resonant dimer antennas can generate a more significant enhancement factor than the arrays of the single cylinder or trimer structures. The enhancement factor is maximized when the *x*-oriented emitter is placed inside the gap (fl = 6.4 × 10^3^), while this enhancement will decrease to fl = 2.8 × 10^3^ when it is above the structure. This behavior is mainly due to the excitation rate enhancement factor.

However, considering a more realistic case, in which the orientation of the emitter is random, an average of the *X*, *Y*, and *Z* orientations of an emitter is performed. The fluorescence enhancement of an emitter above the structure will decrease in the case of cylinder and dimer nanoantennas. However, it will increase when coupled to the trimer structure due to the large increase in the collection efficiency enhancement, which is a result of the *z*-oriented emitter, as shown in Table 1. The same contribution from the *z*-oriented emitter is found when it is on the side of a cylinder structure, which increases the overall fluorescence enhancement to fl = 3.2 × 10^3^.

**Figure 10:**
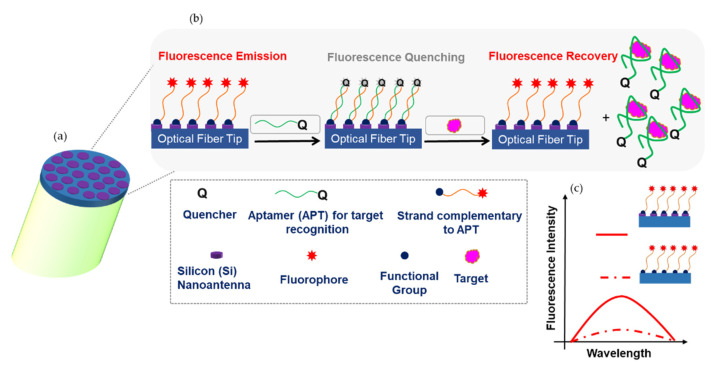
(**a**) Probe’s nanoantenna schematic. (**b**) Proposed detection technique based on the probe’s nanoantenna functionalization with a strand labeled with a fluorophore capable to hybridize with a complementary aptamer bearing a quencher. When the fluorophore and the quencher are in close proximity, fluorescence quenching occurs. Fluorescence is recovered in the presence of the target. (**c**) Illustration of the fluorescence intensity enhancement.

Experimentally, the advanced MS assisted probe with high fluorescence enhancing capability, will be used for sensing biomolecule targets. Figure 10 shows a schematic of the proposed aptamers-based displacement assay, where labeled Aptamers are used for detecting target molecules in solution. In detail, the aptamers design implies that when the labeled strand is attached to the silicon NSs and the recognition aptamer (containing the quencher) hybridizes, the quencher comes in close proximity and fluorescence quenching occurs. In the presence of the target, the specific and strong interaction with the aptamer lead to the aptamer/target complex in the solution. The quencher and the fluorophore are no longer in proximity which results in fluorescence emission recovery related to the target concentration in the solution. The fluorescence intensity is enhanced due to the dielectric NSs found on the fiber tip. This will allow the target detection in a solution with a very low concentration. As the schematic shows, the emitters will be randomly spread around the NS at a distance equal to the length of the used biomolecules. On this basis, we considered the average electric field enhancement inside a square volume surrounding the NS at a maximum distance equal to 15 nm to calculate the overall fluorescence enhancement. In this case, the overall fluorescence enhancement will decrease mainly due to the decrease in the excitation rate enhancement as the distance increases from the nanoantenna. The overall fluorescence enhancement decreased to fl = 144 in case of an array of single cylindrical NS, to fl = 210 in the case of dimer NS (an order of magnitude lower than the case considering lower distances), and to fl = 267 in the case of trimer NS.

### 4.2. Feasibility Study of Fabricating Dielectric MSs on MM Fiber end Face

**Figure 11:**
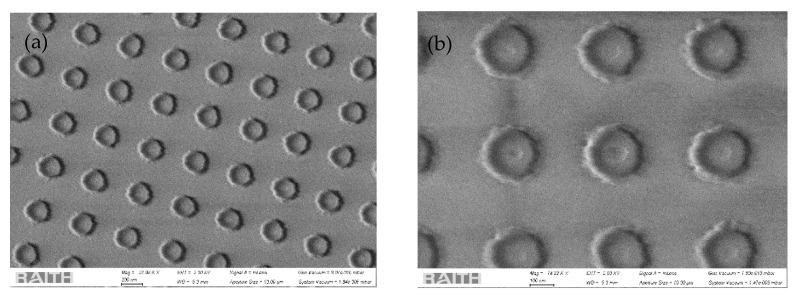
SEM image of cylinder NSs fabricated on a MM fiber tip. (**a**) An overview image of the cylinders array. (**b**) A detailed picture of the realized NSs.

Lithographic methods have been proven to be effective in creating an array of silicon nanoantennas [18,46] on planar substrates. Such a method provides high repeatability and precision in creating complex structures with controlled geometric parameters (diameter, thickness, position). The same process can be adopted to realize the proposed all-dielectric MSs on the tip of standard optical fibers. We start with the less complex structure to be fabricated, i.e., an array of silicon cylinder NSs with a thickness of 120 nm, a diameter of 160 nm, and a period of 448 nm. The NSs were fabricated by means of electron beam lithography (EBL) and reactive ion etching (RIE) on the tip of a MM fiber (FP200ERT) with core and cladding diameter of 200 µm and 225 µm, respectively. The fiber tip is cleaved, then treated with ethanol in an ultrasonic bath to remove any residues and deposited with an a-Si layer of thickness 120 nm via electron beam evaporation. After the deposition of the a-Si layer, a positive tone electron beam resist has been deposited onto the optical fiber tip by means of a customized spin coater, whose rotating plate has been properly drilled to host the fiber tip. A negative mask consisting of a periodic array of holes has been patterned into the resist layer through the EBL Raith 150 system with an acceleration voltage of 10 kV, a numerical aperture of 15 µm and a dose of 130 µC/cm^2^. After the resist development, a 25 nm thick chromium layer has been deposited onto the patterned resist via a DC sputter coater; a properly drilled holder keeps the fiber tip orthogonal to the chromium target. Then, a lift-off process (as reported in [77]) is performed to realize the hard mask that will allow the pattern transfer to the active a-Si layer. The transfer occurs via the Oxford Plasmalab 80 Plus RIE system, with a forward radiofrequency power of 300 W in a mixture of CF_4_ and O_2_ gases with a 50:2 sccm ratio. Finally, the chromium hard mask is removed via a wet etching process. Morphological analysis via a scanning electron microscope (SEM) is performed at every lithographic step in order to evaluate the feasibility of the fabrication process for the proposed design. The first trials show that the dielectric NSs with the selected periodicity, height and diameter are feasible to be fabricated on the tip of MM fibers. As shown in Figure 11, a homogenous array of a-Si cylinders has been successfully fabricated onto a MM fiber tip. The slight difference between the desired cylinder radius and the fabricated one (about ~10 nm) is due to the fabrication tolerance caused by the several fabrication steps. This variation could be reduced by acting on the EBL mask dimensions according to the feasibility of the subsequent fabrication steps. The lateral walls of the cylinders appear slightly sloped due to the underetch effect, which is an intrinsic issue of the RIE process even for small NSs height. To mitigate this effect, a very thin itanium (Ti) layer (about 7 nm) has been interposed between the fiber end-face and the a-Si active layer. Indeed, this metallic layer reduces the CF_4_ ions scattering during the RIE process, allowing the fabrication of NSs with a reasonable shape. We are aware that the presence of Ti might reduce fluorescence enhancement; hence, possible future directions are: (i) to include Ti layer in the optimized numerical procedure; and (ii) to optimize the fabrication technique in order to eliminate the need of the Ti layer.

## 5. Conclusions

In this paper, we laid the basis for the development of plug-and-play LOF optrodes based on the integration of fluorescence-enhancing a-Si MS on the tip of multimode fibers. Specifically, we developed a numerical environment based on FEM analysis to compute the fluorescence enhancement factor provided by a-Si MSs fabricated on the tip of a multimode fiber, which is used both as illuminating and collecting probe. We compared the performance of arrays based on cylindrical a-Si NSs in the single and dimer/trimer configuration fabricated on the Silica substrate of the fiber tip. According to the numerical analysis, the enhancement factor, evaluated as the product of three factors: (i) excitation enhancement; (ii) quantum yield enhancement; (iii) collection efficiency enhancement, was found to be up to three orders of magnitude when the location of the fluorescent emitter is controlled. Furthermore, we envisioned a realistic case study where the advanced LOF probe is used in a realistic scenario in order to evaluate the degradation in the overall fluorescence enhancement when considering the random spread of the labelled fluorophores around the NSs at further distances with unpredictable dye orientation. The feasibility analysis to fabricate the designed MS, based on single Si cylinder, on the termination of multimode optical fibers has been carried out using EBL and RIE techniques, demonstrating that the selected fabrication route enables the correct integration of the nanoantennas. In conclusion, this work presents the guidelines for the design and development of advanced LOF platforms which are expected to reach very high sensitivity, thus yielding a major breakthrough in the development of point of care devices.

## Figures and Tables

**Table 1:** Collection efficiency enhancement of an optical fiber with NA = 0.5. The dipole is located at the center 7 nm above the structure. *X*, *Y*, and *Z* orientations of the dipole are considered.

Dipole Orientation	Array of Cylinders	Array of 20 nm Gap Dimers	Array of Trimers
*X*-oriented dipole	10	8.5	8.24
*Y*-oriented dipole	10	3.29	5.42
*Z*-oriented dipole	0.11	0.11	61.49

**Table 2:** Comparison of the fluorescence enhancements of an emitter coupled with arrays of different geometries, 7 nm above the structure at λ = 650 nm for an *x*-oriented dipole (top row) and averaged over dipole orientation (bottom row).

	Nanostructure	$\gamma_{exc}$	$\frac{\eta }{\eta_{0}}$	$\frac{\kappa_{coll}}{\kappa_{coll}^{0}}$	fl
*X*-Oriented Dipole	Cylinder	17	1.6	10.2	277
	Dimer	170	2	8.5	2890
	Trimer	22	1.5	8.24	271
Averaged-Oriented Dipole	Cylinder	17	1.6	6	163
	Dimer	170	1.5	4	1020
	Trimer	22	1.6	25	880

**Table 3:** Comparison of the fluorescence enhancements of an emitter coupled with arrays of different geometries at λ = 650 nm. The dipole is in the gap of the dimer and trimer and on the side of the cylinder.

	Nanostructure	$\gamma_{exc}$	$\frac{\eta }{\eta_{0}}$	$\frac{\kappa_{coll}}{\kappa_{coll}^{0}}$	fl
*X*-Oriented Dipole	Cylinder	61	1.82	8.28	919
	Dimer	324	2.33	8.55	6454
	Trimer	432	1.89	7.5	6123
Averaged-Oriented Dipole	Cylinder	61	1.62	32.79	3240
	Dimer	324	1.61	3.92	2044
	Trimer	432	1.69	8.04	5869

## Data Availability

Not applicable.
